# Moving forward in treatment of posttraumatic stress disorder: innovations to exposure-based therapy

**DOI:** 10.1080/20008198.2018.1458568

**Published:** 2018-05-18

**Authors:** Mirjam J. Nijdam, Eric Vermetten

**Affiliations:** a Arq Psychotrauma Expert Group, Diemen, The Netherlands; b Department of Psychiatry, Academic Medical Center at the University of Amsterdam, Amsterdam, The Netherlands; c Department of Psychiatry, Leiden University Medical Center, Leiden, The Netherlands; d Military Mental Health Research, Ministry of Defense, Utrecht, The Netherlands

**Keywords:** Posttraumatic stress disorder, psychotherapy, therapy process research, virtual reality treatment, exercise therapy, treatment outcome, treatment resistance, trastorno por estrés postraumático, psicoterapia, investigación del proceso de terapia, tratamiento con realidad virtual, terapia de ejercicio, resultado del tratamiento, resistencia al tratamiento, 创伤后应激障碍, 心理治疗, 治疗过程研究, 虚拟现实治疗, 锻炼治疗, 治疗结果, 治疗阻抗, • The treatment of PTSD can be greatly enhanced by empowering the patient to engage in a multi-modal virtual reality.• The patient is walking (instead of sitting) while being exposed to self-chosen pictures of traumatic hot spots.• This novel approach to treatment of PTSD builds on a cognitive-motor interaction in a virtual interactive environment.• Multimodular Motion-Assisted Memory Desensitization and Reprocessing (3MDR) is based on the theory of memory reconsolidation and the embodiment of cognition.• The patient steps into the past by using forward motion as an essential ingredient to augment the impact of exposure to traumatic events.• Innovations with personalized VR and motion need to be further investigated and implemented in current therapy settings.• These approaches contribute to enhancement of personal efficacy and self-reflectiveness generated by a sense of presence and emotional engagement.

## Abstract

The field of treatment of posttraumatic stress disorder (PTSD) has been a pacesetter for the changing face of psychotherapy, as is illustrated in the introduction of Virtual Reality Exposure Therapy. This paper outlines a novel approach that builds on a cognitive-motor interaction in a virtual interactive environment. It is based on the theory of memory reconsolidation and the embodiment of cognition. The framework we envision allows the patient to 'step into the past' by using forward motion as an essential ingredient to augment the impact of exposure to traumatic events. The behavioural response of approaching that is the exact opposite from the avoidance usually applied by patients and the enhancement of divergent thinking are the most prominent hypothesized mechanisms of action. This can contribute to strengthening of personal efficacy and self-reflection that is generated by high emotional engagement, as well as a sense of accomplishment and enhanced recovery as illustrated by a clinical case example. We argue that innovations with personalized virtual reality and motion need to be further investigated and implemented in current therapy settings.

## Abstract (es)

El campo del tratamiento del trastorno por estrés postraumático (TEPT) ha sido de los más avanzados en cuanto a los cambios que han tenido lugar en la psicoterapia, como la introducción de la Terapia de Exposición con Realidad Virtual. Este documento describe un enfoque novedoso que se basa en una interacción cognitivo-motora en un entorno virtual interactivo. Se basa en la teoría de la reconsolidación de la memoria y la materialización de la cognición. El marco de trabajo que visualizamos le permite al paciente literalmente entrar en el pasado usando el movimiento hacia adelante como un ingrediente esencial para aumentar el impacto de la exposición a eventos traumáticos. La respuesta conductual de aproximación que es exactamente lo opuesto a la evitación que generalmente aplican los pacientes y la mejora del pensamiento divergente son los mecanismos de acción hipotéticos más prominentes. Esto puede contribuir al fortalecimiento de la eficacia personal y la autorreflexión que se genera por una alta implicación emocional, así como una sensación de logro y una recuperación mejorada como muestra un ejemplo de caso clínico. Argumentamos que las innovaciones con VR y movimiento personalizados deben investigarse más a fondo y aplicarse en los entornos terapéuticos actuales.

## Abstract (zh)

在心理治疗的变革中，创伤后应激障碍（PTSD）属于领跑者，虚拟现实暴露疗法的引入就是这种情况。本文概述了一种基于虚拟交互环境中的认知 - 运动交互的新颖方法。它是基于记忆再巩固和具身认知理论。我们设想的框架允许患者通过使用向前运动作为增加暴露于创伤事件的影响的基本成分，真实地‘进入’过去。向前靠近的行为反应与患者通常采用的回避和发散性思维的加强完全相反，这是行为最显著的假设机制。这可以有助于加强由高度情绪交互产生的个人效能感和自我反思，以及如一个临床案例所示的成就感和强化康复。我们提出，具有个性化VR和动作的创新需要在当前的治疗设置中得到进一步研究和实施。

## 1. Introduction

In the history of psychotherapy the position of the patient in therapy has seen some remarkable shifts. In analytic therapies, the patient was lying on a couch and the therapist was sitting behind the patient. In order to improve communication, this changed to sitting with a face-to-face contact for both. More recently, the sitting position is challenged in the light of the opportunities that emerge in the employment of virtual reality (VR), as well as the opportunity for movement (walking) and interaction. The shifts have always been in the interest of improvement of the effectiveness of therapy. The latest repositioning may be the interactive walking patient. This may be particularly relevant for posttraumatic stress disorder (PTSD) and comes timely since for some groups of patients, such as veterans with PTSD, treatment efficacy is still not optimal and up to two-thirds of them retain their diagnosis after evidence-based treatment (Steenkamp, Litz, Hoge, & Marmar, 2015).

New psychotherapies need to emerge that challenge traditional conventions and configurations. We outline here one of the new treatments in which the traditional chair is replaced by a treadmill and in which the patient participates in the therapeutic process while walking in a personalized VR. With VR technology and use of self-chosen pictures, the approach is precise, moves beyond the ‘one size fits all’ principle, facilitates empowerment, and bypasses cognitive avoidance. In this paper we give an account of enhancement of memory reconsolidation, creativity, and embodiment of cognition by walking. This essay recognizes the value of active participation in treatment for PTSD, with the employment of low level technology using personalized ingredients that aim to improve treatment outcome for a wide range of trauma survivors (Hoge et al., 2014; Van Gelderen, Nijdam & Vermetten, 2018). It sets a framework for a novel approach to delivering psychotherapy to patients with PTSD (https://www.youtube.com/watch?time_continue=4&v=IUnWe7tfgSQ).

## 2. Cognitive-motor interaction in exposure treatment

Walking is defined as guided movement, typically in a forward direction. Walking is one of the most important developmental stages of humans; other modalities of moving forward such as cycling or ice-skating are acquired during later stages. While walking or cycling we barely notice the coordinated neural networks that are required for balance and direction. In cognitive science, the extent to which sensorimotor processes are involved in cognition is extensively studied (Loeffler, Raab, & Canal-Bruland, 2016). Motricity and cognition are functionally connected and both evolved in parallel and independently. Walking has been demonstrated to rely on cognitive-motor interactions that facilitate problem solving (Leisman, Moustafa, & Shafir, 2016), relate to embodiment (Masson, 2015), creativity (Oppezzo & Schwartz, 2014), and may possibly bypass cognitive avoidance by changing the vantage perspective (Williams & Moulds, 2007). These are elements that are critical for the treatment of fear-related and traumatic memories.

Another way in which walking or other forms of movement can work synergistically with treatment of fear-laden or traumatic memories is through release of a series of chemicals (e.g. endogenous opioids, effects on NMDA receptors and HPA axis) among which brain-derived neurotrophic factor (BNDF) may play an important role. From that viewpoint, movement should precede exposure to traumatic memories for an optimal effect. Movement has been shown to lead to better retention of fear extinction (Siette, Reichelt, & Westbrook, 2014). Powers and colleagues tested this effect in humans in a pilot study in which nine patients with a diagnosis of PTSD were randomly assigned to exercise before prolonged exposure treatment or prolonged exposure alone (Powers et al., 2015). The group of patients that exercised on a treadmill before their trauma treatment showed a significantly greater improvement in their PTSD symptoms and significantly increased brain derived neurotrophic factor (BDNF) in comparison to the patients who received exposure alone. BNDF is associated with synaptic plasticity, which is essential for long-term learning and memory. Memory proved to be an important predictor for treatment success in trauma-focused psychotherapy (Nijdam, De Vries, Gersons, & Olff, 2015), thus if exercise and subsequent BDNF release paves the way for increased learning, greater benefit from the intervention could result. If a trauma survivor can benefit in a greater way, this may also enhance the long-term effects drawn from the intervention.

## 3. Walking and talking during exposure in a virtual interactive environment

**Figure 1.**
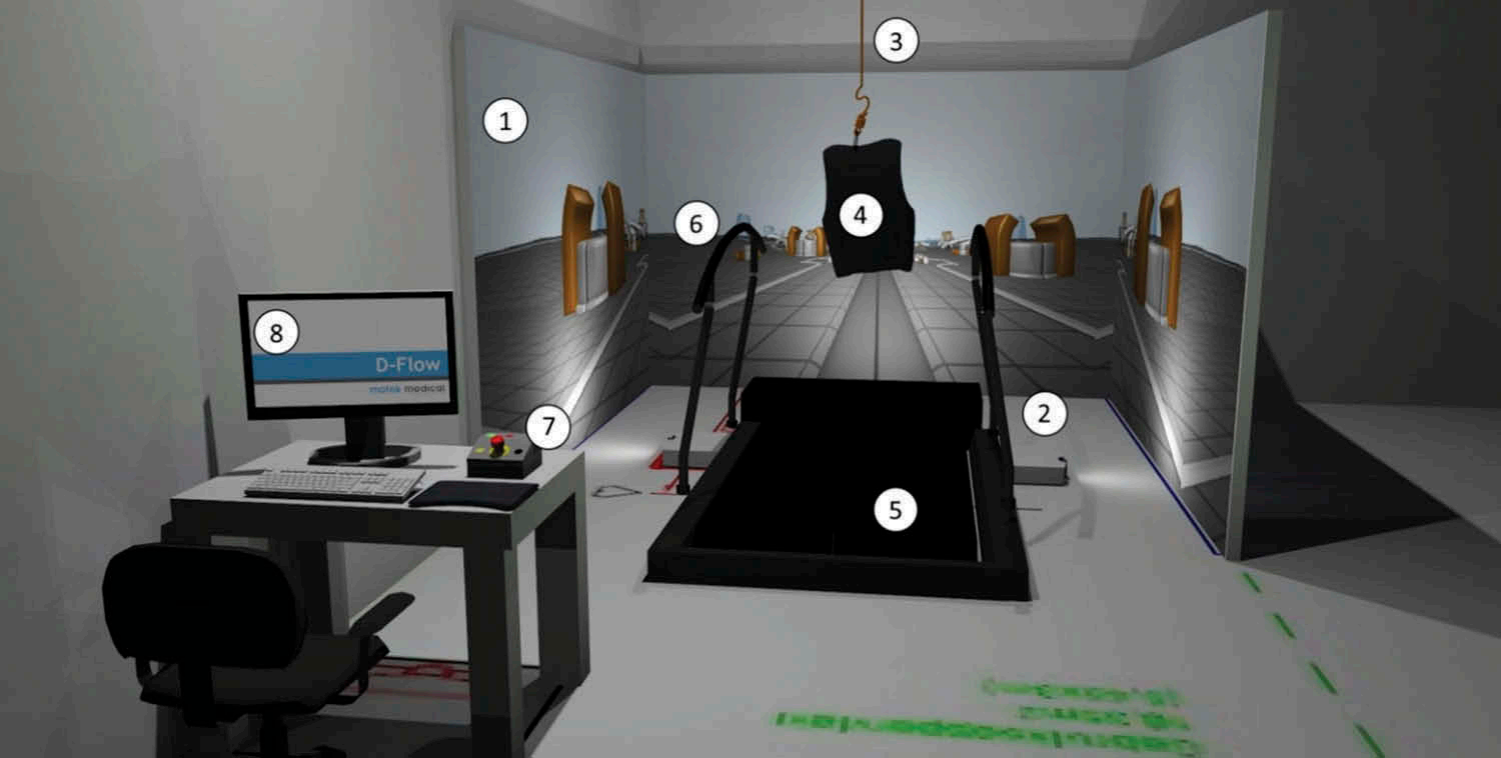
The 3MDR installation. 1. Three projection screens; 2. Three projectors; 3. Ceiling mount and safety line for safety harness; 4. Safety harness; 5. Treadmill; 6. Safety handrails; 7. Emergency stop button and circuit; 8. Desktop PC with operating system.

Benefits of cognitive-motor interaction can also be derived from interventions in which walking and imaginal exposure to traumatic memories are combined at the same time. Through working with amputee soldiers in virtual environments learning to walk again, a collaboration between a psychiatrist and a rehabilitation physician led to an innovative intervention for PTSD. In this treatment, the trauma survivor walks during the entire session. This has been named 3MDR: Multimodular Motion-assisted Memory Desensitization and Reprocessing (Vermetten, Meijer, van der Wurff, & Mert, 2013). Patients walk in a treadmill towards self-chosen, high affect pictures of their deployment (see Figure 1). 2012,2014The behavioral response of approachment (‘moving towards’) (Badour, Boningen etc.) is quite the opposite from avoidance (‘moving away’) that typically is used. Together with effect on divergent thinking (Opezzo and Schwartz, 2014) these are the key drivers for our approach. Taken together, these make it plausible that patients can more easily access other associations related to their trauma memory while walking (see Clinical case). Other known efficacious components originating from Virtual Reality Exposure Therapy (VRET) and Eye Movement Desensitization and Reprocessing therapy (EMDR) complete the treatment. The virtual reality environment in this treatment enhances the sense of ‘presence’ (Riva, Banos, Botella, Mantovani, & Gaggioli, 2016) and often leads to in-session time distortion, as is typical with negative pictures and a low experienced level of control (Mereu & Lleras, 2013). The task assignment is such that when the trauma survivor has fully narrated what the picture has evoked in terms of emotions and thoughts, a dual task follows with the instruction to focus on all that has come up and at the same time follow a moving ball on the screen with their eyes and mention the changing numbers in the ball. The trauma survivor continues to walk, so these combined interventions could be termed a ‘triple task’ to perform. Another variant of triple task taxation during trauma treatment is doing exposure or EMDR treatment while both patient and therapist are on hometrainers. In this way, the motion element contributes to desensitization of the trauma memory by way of working memory taxation (Gunter and Bodner, 2008).

## 4. Clinical case

John is a 51 year old veteran with chronic PTSD. From a couple of months after his last deployment to Afghanistan in 2010 he started to suffer from symptoms of cluster headaches as well as symptoms of PTSD. During his last deployment working on the air strip in Kandahar his role was to mount bombs on F16 airplanes and secure their operability. He needed to be extremely precise in his work. He was sensitized by missiles that were frequently launched from outside into the base and was exposed to traumatic events in which he was close to death. Upon return he started suffering from flashbacks, continuation of hyperarousal, restlessness and exaggerated startle. He was unable to wind down, became irritable, and slept poorly. He had been taking high doses of medication for the cluster headaches. These symptoms, together with his frequent headaches, led to diagnosis of cluster headaches and PTSD and forced him to discontinue from work and take early retirement after 30 years of service. He was easily startled, unable to express emotions, and could not grieve over his forced retirement. Although he was motivated to therapy his behavioural and cognitive avoidance prevented any success with exposure-based interventions. After EMDR and cognitive behavioural therapy with some hesitance he consented to 3MDR. He was able to tolerate exposure to pictures serving as triggers for traumatic memories that he had greatly suppressed and avoided talking about. This resulted in session-by-session decreased startle and a remarkable increased openness to explore grief. He was surprised to feel how strenuous the sessions were as well by his frank and open emotional responses. He was afraid that guilt would emerge which it did not. He noticed how over six sessions his startle and fear shifted to anger, and his grief shifted to pride. He attributed these shifts to walking into the virtual environment as well as the release of affect that he now was able to experience. He felt empowered to further explore these emotions, and noticed how his sleep improved as well as the return of positive emotions and memories of his deployment he had been suppressing. The therapy boosted his self-confidence, and he felt empowered in the maintenance of the cluster migraines. His spouse was happy to see a little more of the ‘old John’ back in the house.

## 5. Road map for further research

Different forms of treatment are now available in which exercise is an adjunct to treatment for PTSD in line with recent developments in other psychiatric disorders (Jacquart et al., 2014). Some forms presume that exercise should precede exposure to trauma memories, whereas others see advantages in combining the different elements at the same time. Remarkable in this new wave of treatments is that exercise is coupled to exposure rather than used as a standalone intervention because of the hypothesized synergistic effect. Physical activity based interventions alone were shown to have small- to medium-sized effects in a meta-analysis (Rosenbaum et al., 2015), but the effect can be much larger when combined with trauma exposure.

Next to methodologically sound randomized trials to investigate the effects of these innovative interventions on a sufficiently large scale, it is necessary to investigate the working mechanisms of these combined interventions experimentally. A number of possible explanations could be investigated in experiments with clear, testable hypotheses. First, the chemical output of exercise or movement can be beneficial in processing traumatic memories in a number of ways. Second, a trauma memory could fade more effectively by performing multiple tasks (Gunter & Bodner, 2008), including movements, that tax working memory. Third, by means of embodied cognition and increased creativity (Oppezzo & Schwartz, 2014), moving could influence the content of the thought processes about the trauma and lead to different associations and changed orientations. Fourth, walking could be a means to get rid of the tension exposure evokes in the body. In conducting these studies, one should bear in mind that the findings are not necessarily mutually exclusive. Another important issue for the research agenda is to investigate timing, intensity, and type of movement to determine the optimal effect in combination with trauma exposure.

## 6. Conclusion

While the effects of movement components and interaction with virtual reality in the treatment of PTSD still need to be rigorously and systematically studied, a third wave of treatment enhancers is already providing additional competitive and novel opportunities. The playfulness and gamification approach in home based devices and computer games offers yet another domain that can provide a supportive platform to therapy. Similar to the way augmented realities are currently used in treatment, aspects of these ‘new worlds’ may be combined with psychotherapy to further sustain a fresh, timely, and personalized experience of treatment (Riva et al., 2016). These approaches contribute to enhancement of personal efficacy and self-reflectiveness generated by a sense of presence and emotional engagement. We believe that these new opportunities should be further explored and offered with priority to patients with PTSD who belong to populations that have historically shown suboptimal effects in trauma treatment. As drastically as these interventions may change the traditional therapeutic constellation in the near future, we deem it most important to investigate their potential to optimize ways to symptom reduction and increase quality of life in our patients.
